# 2-(4-Chloro­phen­yl)-2-oxoethyl 4-methyl­benzoate

**DOI:** 10.1107/S1600536811042851

**Published:** 2011-10-22

**Authors:** Hoong-Kun Fun, Wan-Sin Loh, B. Garudachari, Arun M. Isloor, M. N. Satyanarayana

**Affiliations:** a X-ray Crystallography Unit, School of Physics, Universiti Sains Malaysia, 11800 USM, Penang, Malaysia; b Organic Electronics Division, Department of Chemistry, National Institute of Technology-Karnataka, Surathkal, Mangalore 575 025, India; c Department of Physics, National Institute of Technology-Karnataka, Surathkal, Mangalore 575 025, India

## Abstract

In the title compound, C_16_H_13_ClO_3_, the dihedral angle between the benzene rings is 80.74 (8)°. In the crystal, C—H⋯O hydrogen bonds link the mol­ecules to form *C*(11) chains propagating in [010].

## Related literature

For a related structure and background references to phenacyl benzoates, see: Fun *et al.* (2011 ▶).
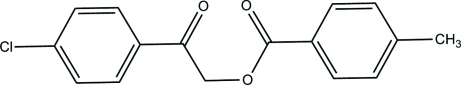


## Experimental

### 

#### Crystal data

C_16_H_13_ClO_3_
*M* *_r_* = 288.71
Monoclinic, 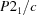

*a* = 5.9132 (4) Å
*b* = 8.5044 (6) Å
*c* = 27.8767 (18) Å
β = 95.880 (1)°
*V* = 1394.49 (16) Å^3^
*Z* = 4
Mo *K*α radiation
μ = 0.28 mm^−1^
*T* = 297 K
0.51 × 0.30 × 0.06 mm

#### Data collection

Bruker SMART APEXII DUO CCD diffractometer
Absorption correction: multi-scan (*SADABS*; Bruker, 2009 ▶) *T*_min_ = 0.871, *T*_max_ = 0.983
21275 measured reflections
4070 independent reflections
2628 reflections with *I* > 2σ(*I*)
*R*_int_ = 0.027

#### Refinement

*R*[*F* ^2^ > 2σ(*F* ^2^)] = 0.046
*wR*(*F* ^2^) = 0.144
*S* = 1.05
4070 reflections
182 parameters
H-atom parameters constrained
Δρ_max_ = 0.20 e Å^−3^
Δρ_min_ = −0.42 e Å^−3^

### 

Data collection: *APEX2* (Bruker, 2009 ▶); cell refinement: *SAINT* (Bruker, 2009 ▶); data reduction: *SAINT*; program(s) used to solve structure: *SHELXTL* (Sheldrick, 2008 ▶); program(s) used to refine structure: *SHELXTL*; molecular graphics: *SHELXTL*; software used to prepare material for publication: *SHELXTL*, *PLATON* (Spek, 2009 ▶).

## Supplementary Material

Crystal structure: contains datablock(s) global, I. DOI: 10.1107/S1600536811042851/hb6454sup1.cif

Structure factors: contains datablock(s) I. DOI: 10.1107/S1600536811042851/hb6454Isup2.hkl

Supplementary material file. DOI: 10.1107/S1600536811042851/hb6454Isup3.cml

Additional supplementary materials:  crystallographic information; 3D view; checkCIF report

## Figures and Tables

**Table 1:** Hydrogen-bond geometry (Å, °)

*D*—H⋯*A*	*D*—H	H⋯*A*	*D*⋯*A*	*D*—H⋯*A*
C16—H16*C*⋯O1^i^	0.96	2.46	3.383 (2)	162

Symmetry code: (i) 
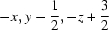
.

**Fig. 1.**
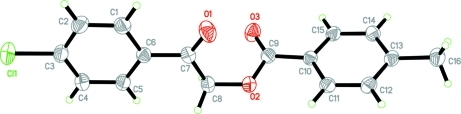
The molecular structure of the title compound, showing 30% probability displacement ellipsoids.

**Fig. 2.**
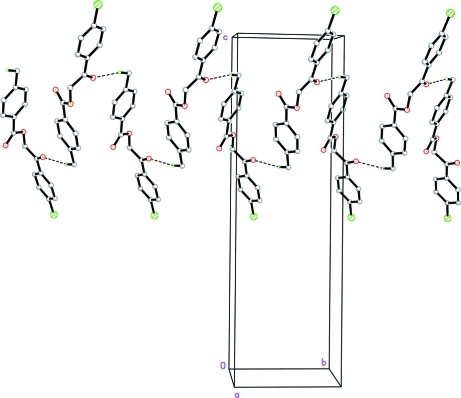
The crystal packing of the title compound, viewed along the showing the a axis. H atoms not involved in the intermolecular interactions (dashed lines) have been omitted for clarity.

## supplementary crystallographic information

### Comment

As part of our ongoing studies of phenacyl benzoates (Fun *et al.*, 2011), we now report the synthesis and sturcture of the title compound, (I).

In the title compound (Fig. 1), the dihedral angle formed between the chloro-substituted (C1–C6) and the methyl-substituted (C10–C15) benzene rings is 80.74 (8)°. Bond lengths and angles are within the normal ranges and are comparable to a related structure (Fun *et al.*, 2011).

In the crystal (Fig. 2), intermolecular C16—H16C···O1 hydrogen bonds (Table 1) link the molecules to form chains along the *b* axis.

### Experimental

A mixture of 4-methylbenzoic acid (1.0 g, 0.0073 mol), potassium carbonate (1.10 g, 0.0080 mol) and 2-bromo-1-(4-chlorophenyl)ethanone (1.70 g, 0.0073 mol) in dimethylformamide (10 ml) was stirred at room temperature for 2 h. On cooling, colourless needle-shaped crystals of the title compound began to separate out.  They were collected by filtration and recrystallized from ethanol to yield colourless plates of (I). Yield: 1.95 g, 92.8%. *M. p*: 405–406 K.

### Refinement

All H atoms were positioned geometrically and refined with a riding model with *U*_iso_(H) = 1.2 or 1.5 *U*_eq_(C) [C–H = 0.93 or 0.97 Å]. A rotating group model was applied to the methyl group.

### Crystal data

**Table:**

C_16_H_13_ClO_3_	*F*(000) = 600
*M**_r_* = 288.71	*D*_x_ = 1.375 Mg m^−^^3^
Monoclinic, *P*2_1_/*c*	Mo *K*α radiation, λ = 0.71073 Å
Hall symbol: -P 2ybc	Cell parameters from 4428 reflections
*a* = 5.9132 (4) Å	θ = 2.8–28.1°
*b* = 8.5044 (6) Å	µ = 0.28 mm^−^^1^
*c* = 27.8767 (18) Å	*T* = 297 K
β = 95.880 (1)°	Plate, colourless
*V* = 1394.49 (16) Å^3^	0.51 × 0.30 × 0.06 mm
*Z* = 4	

### Data collection

**Table:**

Bruker SMART APEXII DUO CCD diffractometer	4070 independent reflections
Radiation source: fine-focus sealed tube	2628 reflections with *I* > 2σ(*I*)
graphite	*R*_int_ = 0.027
φ and ω scans	θ_max_ = 30.1°, θ_min_ = 1.5°
Absorption correction: multi-scan (*SADABS*; Bruker, 2009)	*h* = −8→8
*T*_min_ = 0.871, *T*_max_ = 0.983	*k* = −11→12
21275 measured reflections	*l* = −39→39

### Refinement

**Table:**

Refinement on *F*^2^	Primary atom site location: structure-invariant direct methods
Least-squares matrix: full	Secondary atom site location: difference Fourier map
*R*[*F*^2^ > 2σ(*F*^2^)] = 0.046	Hydrogen site location: inferred from neighbouring sites
*wR*(*F*^2^) = 0.144	H-atom parameters constrained
*S* = 1.05	*w* = 1/[σ^2^(*F*_o_^2^) + (0.0587*P*)^2^ + 0.3124*P*] where *P* = (*F*_o_^2^ + 2*F*_c_^2^)/3
4070 reflections	(Δ/σ)_max_ = 0.002
182 parameters	Δρ_max_ = 0.20 e Å^−^^3^
0 restraints	Δρ_min_ = −0.42 e Å^−^^3^

### Special details

**Table:**

Geometry. All e.s.d.'s (except the e.s.d. in the dihedral angle between two l.s. planes) are estimated using the full covariance matrix. The cell e.s.d.'s are taken into account individually in the estimation of e.s.d.'s in distances, angles and torsion angles; correlations between e.s.d.'s in cell parameters are only used when they are defined by crystal symmetry. An approximate (isotropic) treatment of cell e.s.d.'s is used for estimating e.s.d.'s involving l.s. planes.
Refinement. Refinement of *F*^2^ against ALL reflections. The weighted *R*-factor *wR* and goodness of fit *S* are based on *F*^2^, conventional *R*-factors *R* are based on *F*, with *F* set to zero for negative *F*^2^. The threshold expression of *F*^2^ > σ(*F*^2^) is used only for calculating *R*-factors(gt) *etc*. and is not relevant to the choice of reflections for refinement. *R*-factors based on *F*^2^ are statistically about twice as large as those based on *F*, and *R*- factors based on ALL data will be even larger.

### Fractional atomic coordinates and isotropic or equivalent isotropic displacement parameters (Å^2^)

**Table:**

	*x*	*y*	*z*	*U*_iso_*/*U*_eq_	
Cl1	1.34410 (11)	0.75388 (9)	1.047675 (19)	0.0935 (2)	
O1	0.5306 (2)	0.69016 (17)	0.86404 (5)	0.0739 (4)	
O2	0.6246 (2)	0.50027 (17)	0.79276 (4)	0.0612 (3)	
O3	0.3791 (2)	0.35541 (15)	0.83027 (4)	0.0643 (3)	
C1	0.8059 (3)	0.7539 (2)	0.94970 (7)	0.0569 (4)	
H1A	0.6646	0.8027	0.9453	0.068*	
C2	0.9496 (4)	0.7851 (2)	0.99053 (7)	0.0646 (5)	
H2A	0.9056	0.8539	1.0138	0.078*	
C3	1.1586 (3)	0.7138 (2)	0.99658 (6)	0.0589 (4)	
C4	1.2267 (3)	0.6099 (2)	0.96302 (6)	0.0598 (4)	
H4A	1.3684	0.5617	0.9678	0.072*	
C5	1.0808 (3)	0.5784 (2)	0.92215 (6)	0.0530 (4)	
H5A	1.1248	0.5083	0.8992	0.064*	
C6	0.8693 (3)	0.65035 (18)	0.91501 (5)	0.0462 (3)	
C7	0.7093 (3)	0.62133 (19)	0.87099 (6)	0.0487 (4)	
C8	0.7785 (3)	0.5032 (2)	0.83558 (6)	0.0579 (4)	
H8A	0.9298	0.5283	0.8273	0.070*	
H8B	0.7842	0.3997	0.8503	0.070*	
C9	0.4256 (3)	0.42441 (19)	0.79494 (6)	0.0484 (4)	
C10	0.2774 (3)	0.43770 (17)	0.74889 (5)	0.0438 (3)	
C11	0.3350 (3)	0.53418 (19)	0.71169 (6)	0.0496 (4)	
H11A	0.4730	0.5876	0.7146	0.060*	
C12	0.1863 (3)	0.55014 (19)	0.67042 (6)	0.0523 (4)	
H12A	0.2264	0.6144	0.6456	0.063*	
C13	−0.0210 (3)	0.47287 (18)	0.66499 (5)	0.0482 (4)	
C14	−0.0734 (3)	0.3726 (2)	0.70156 (6)	0.0531 (4)	
H14A	−0.2093	0.3166	0.6982	0.064*	
C15	0.0749 (3)	0.3551 (2)	0.74308 (6)	0.0505 (4)	
H15A	0.0379	0.2873	0.7672	0.061*	
C16	−0.1878 (3)	0.5006 (2)	0.62043 (6)	0.0604 (4)	
H16A	−0.1102	0.4892	0.5920	0.091*	
H16B	−0.2492	0.6049	0.6215	0.091*	
H16C	−0.3089	0.4252	0.6197	0.091*	

### Atomic displacement parameters (Å^2^)

**Table:**

	*U*^11^	*U*^22^	*U*^33^	*U*^12^	*U*^13^	*U*^23^
Cl1	0.0873 (4)	0.1316 (6)	0.0578 (3)	−0.0190 (4)	−0.0114 (3)	−0.0225 (3)
O1	0.0601 (8)	0.0737 (8)	0.0831 (9)	0.0200 (7)	−0.0156 (7)	−0.0148 (7)
O2	0.0487 (6)	0.0890 (9)	0.0452 (6)	−0.0116 (6)	0.0008 (5)	−0.0060 (6)
O3	0.0746 (8)	0.0656 (8)	0.0513 (7)	−0.0104 (7)	−0.0006 (6)	0.0087 (6)
C1	0.0517 (9)	0.0576 (10)	0.0617 (10)	0.0007 (8)	0.0071 (8)	−0.0088 (8)
C2	0.0714 (12)	0.0683 (11)	0.0555 (10)	−0.0103 (10)	0.0130 (9)	−0.0163 (8)
C3	0.0615 (10)	0.0705 (11)	0.0438 (8)	−0.0165 (9)	0.0011 (7)	−0.0034 (8)
C4	0.0510 (9)	0.0736 (11)	0.0526 (9)	0.0014 (8)	−0.0044 (7)	−0.0015 (8)
C5	0.0518 (9)	0.0582 (9)	0.0480 (8)	0.0033 (7)	0.0011 (7)	−0.0065 (7)
C6	0.0466 (8)	0.0462 (8)	0.0457 (8)	−0.0043 (6)	0.0039 (6)	−0.0001 (6)
C7	0.0452 (8)	0.0482 (8)	0.0518 (8)	−0.0021 (7)	−0.0002 (7)	0.0017 (7)
C8	0.0470 (9)	0.0747 (11)	0.0503 (9)	0.0005 (8)	−0.0042 (7)	−0.0102 (8)
C9	0.0497 (8)	0.0485 (8)	0.0469 (8)	0.0006 (7)	0.0046 (7)	−0.0075 (7)
C10	0.0455 (8)	0.0443 (7)	0.0418 (7)	−0.0007 (6)	0.0059 (6)	−0.0054 (6)
C11	0.0483 (8)	0.0488 (8)	0.0522 (8)	−0.0082 (7)	0.0071 (7)	0.0001 (7)
C12	0.0617 (10)	0.0492 (8)	0.0467 (8)	−0.0037 (7)	0.0081 (7)	0.0046 (7)
C13	0.0532 (9)	0.0478 (8)	0.0431 (8)	0.0039 (7)	0.0030 (6)	−0.0083 (6)
C14	0.0483 (8)	0.0598 (10)	0.0508 (8)	−0.0109 (7)	0.0040 (7)	−0.0052 (7)
C15	0.0518 (9)	0.0555 (9)	0.0449 (8)	−0.0097 (7)	0.0082 (7)	0.0004 (7)
C16	0.0638 (11)	0.0631 (10)	0.0524 (9)	0.0048 (9)	−0.0030 (8)	−0.0047 (8)

### Geometric parameters (Å, °)

**Table:**

Cl1—C3	1.7400 (17)	C8—H8A	0.9700
O1—C7	1.2062 (19)	C8—H8B	0.9700
O2—C9	1.349 (2)	C9—C10	1.483 (2)
O2—C8	1.4250 (18)	C10—C15	1.383 (2)
O3—C9	1.203 (2)	C10—C11	1.392 (2)
C1—C2	1.375 (2)	C11—C12	1.381 (2)
C1—C6	1.387 (2)	C11—H11A	0.9300
C1—H1A	0.9300	C12—C13	1.385 (2)
C2—C3	1.371 (3)	C12—H12A	0.9300
C2—H2A	0.9300	C13—C14	1.388 (2)
C3—C4	1.377 (3)	C13—C16	1.523 (2)
C4—C5	1.383 (2)	C14—C15	1.387 (2)
C4—H4A	0.9300	C14—H14A	0.9300
C5—C6	1.388 (2)	C15—H15A	0.9300
C5—H5A	0.9300	C16—H16A	0.9600
C6—C7	1.491 (2)	C16—H16B	0.9600
C7—C8	1.495 (2)	C16—H16C	0.9600
			
C9—O2—C8	117.12 (13)	O3—C9—O2	122.98 (15)
C2—C1—C6	120.75 (17)	O3—C9—C10	125.56 (15)
C2—C1—H1A	119.6	O2—C9—C10	111.46 (14)
C6—C1—H1A	119.6	C15—C10—C11	119.15 (14)
C3—C2—C1	119.28 (17)	C15—C10—C9	119.38 (14)
C3—C2—H2A	120.4	C11—C10—C9	121.45 (14)
C1—C2—H2A	120.4	C12—C11—C10	119.66 (15)
C2—C3—C4	121.52 (16)	C12—C11—H11A	120.2
C2—C3—Cl1	119.91 (15)	C10—C11—H11A	120.2
C4—C3—Cl1	118.57 (15)	C11—C12—C13	121.67 (15)
C3—C4—C5	118.90 (17)	C11—C12—H12A	119.2
C3—C4—H4A	120.5	C13—C12—H12A	119.2
C5—C4—H4A	120.5	C12—C13—C14	118.22 (14)
C4—C5—C6	120.60 (16)	C12—C13—C16	120.48 (15)
C4—C5—H5A	119.7	C14—C13—C16	121.28 (15)
C6—C5—H5A	119.7	C15—C14—C13	120.59 (15)
C1—C6—C5	118.94 (15)	C15—C14—H14A	119.7
C1—C6—C7	118.97 (15)	C13—C14—H14A	119.7
C5—C6—C7	122.08 (14)	C10—C15—C14	120.61 (15)
O1—C7—C6	121.57 (15)	C10—C15—H15A	119.7
O1—C7—C8	120.98 (15)	C14—C15—H15A	119.7
C6—C7—C8	117.46 (13)	C13—C16—H16A	109.5
O2—C8—C7	111.72 (14)	C13—C16—H16B	109.5
O2—C8—H8A	109.3	H16A—C16—H16B	109.5
C7—C8—H8A	109.3	C13—C16—H16C	109.5
O2—C8—H8B	109.3	H16A—C16—H16C	109.5
C7—C8—H8B	109.3	H16B—C16—H16C	109.5
H8A—C8—H8B	107.9		
			
C6—C1—C2—C3	0.6 (3)	C8—O2—C9—O3	−3.1 (2)
C1—C2—C3—C4	−0.9 (3)	C8—O2—C9—C10	176.99 (14)
C1—C2—C3—Cl1	179.20 (14)	O3—C9—C10—C15	−5.7 (2)
C2—C3—C4—C5	0.6 (3)	O2—C9—C10—C15	174.20 (14)
Cl1—C3—C4—C5	−179.55 (14)	O3—C9—C10—C11	172.90 (16)
C3—C4—C5—C6	0.1 (3)	O2—C9—C10—C11	−7.2 (2)
C2—C1—C6—C5	0.0 (3)	C15—C10—C11—C12	2.2 (2)
C2—C1—C6—C7	−179.28 (16)	C9—C10—C11—C12	−176.38 (15)
C4—C5—C6—C1	−0.4 (3)	C10—C11—C12—C13	0.3 (3)
C4—C5—C6—C7	178.91 (16)	C11—C12—C13—C14	−2.6 (2)
C1—C6—C7—O1	2.9 (3)	C11—C12—C13—C16	176.20 (15)
C5—C6—C7—O1	−176.38 (18)	C12—C13—C14—C15	2.3 (2)
C1—C6—C7—C8	−177.07 (16)	C16—C13—C14—C15	−176.49 (15)
C5—C6—C7—C8	3.6 (2)	C11—C10—C15—C14	−2.5 (2)
C9—O2—C8—C7	−77.7 (2)	C9—C10—C15—C14	176.13 (15)
O1—C7—C8—O2	7.0 (3)	C13—C14—C15—C10	0.2 (3)
C6—C7—C8—O2	−173.03 (14)		

### Hydrogen-bond geometry (Å, °)

**Table:**

*D*—H···*A*	*D*—H	H···*A*	*D*···*A*	*D*—H···*A*
C16—H16C···O1^i^	0.96	2.46	3.383 (2)	162

Symmetry codes: (i) −*x*, *y*−1/2, −*z*+3/2.
